# Whither verbal autopsy?

**DOI:** 10.1186/1478-7954-9-23

**Published:** 2011-08-01

**Authors:** Peter Byass

**Affiliations:** 1 Umeå Centre for Global Health Research, Umeå University, 90187 Umeå, Sweden; 2 MRC/Wits Rural Public Health and Health Transitions Research Unit (Agincourt), School of Public Health, Faculty of Health Sciences, University of the Witwatersrand, Johannesburg, South Africa

## Commentary

Wherever the field of verbal autopsy (VA) may be heading, the exciting and considerable extent of new work presented in this *Population Health Metrics *series clearly shows that the topic is not withering. The Global Congress on Verbal Autopsy held in Bali in February 2011 undoubtedly marked a significant milestone: VA has come of age as an area of scientific interest in its own right. We may, however, be at something of a tipping point in that most of the work over the past few decades has (perhaps largely unconsciously) concentrated on presenting VA (usually interpreted by physicians) as a second-best substitute for medical certification of cause of death, particularly for application in areas where routine certification is either practiced selectively or not required [1]. However, it now emerges that medical certification of death is not as reliable as is often assumed, and physicians are also not particularly good at interpreting VA data consistently and reliably [2]. We have also learned that evaluations of cause-specific mortality are generally compromised by a lack of true gold standard data and metrics for comparative purposes [3,4]. At the same time, the dominance of research domains in VA applications is partly giving way to concepts of using VA in more routine ways, at least as an interim strategy in countries where universal routine death certification remains some way off. These perceived needs, coupled with new methodological developments, offer exciting prospects.

The VA literature has extensively used and abused the concept of "gold standards" for validating cause of death determination. Metallurgists would say that 100% pure gold is an impossibility; the highest possible quality is normally certified as being 99.9% gold, while most of the quality-assured gold we encounter on an everyday basis ranges from 37% to 75% purity. It is perhaps also worth reflecting that 99% pure gold is an extremely soft and somewhat impractical material. Cause of death, on the spectrum of measurable biomedical phenomena, is also a somewhat soft commodity. For that reason, any approach to assessing cause of death involves alloying professional expertise with the best evidence in order to generate robust outcomes. Different approaches to cause of death determination do this in different ways. Pathologists undertaking autopsies combine their specific expertise with visualized intracorporeal evidence to arrive at a cause of death (which frequently varies from a nonautopsy cause of death [5,6]). Physicians certifying a patient's death combine their expertise with antemortem data, the quality and extent of which may vary considerably. Verbal autopsy interpreted by physicians relies on similar expertise to medical certification, but using the very different evidence base of the VA interview. Modeled approaches to cause of death determination need some kind of expert input - whether it be, for example, the physician committee that established the mapping between clinical criteria and causes of death in the new Population Health Metrics Research Consortium (PHMRC) dataset [3] or the expert group that refined prior probability estimates in the InterVA model [7] - and to incorporate that captured expertise with available evidence to deliver a reliable model.

As in any field of science, methods for cause of death determination evolve and develop over time. Any "good" approach ideally needs to demonstrate both a satisfactory quantitative metric of performance and established widespread confidence among its users. In this respect the ground is currently somewhat unstable; the widespread confidence in physician-derived cause of death is being challenged, and InterVA, the cause of death model that has been most widely applied during the past decade, has so far primarily established its performance against physicians [8]. New ideas for models may perform well in terms of quantitative metrics against test datasets [4] but as yet have not achieved widespread confidence among actual users. The future for VA is therefore likely to be dynamic and exciting - and will hopefully help the world to move to a position where mortality patterns are well documented and available as evidence to feed into health service planning.

## Competing interests

PB is a member of the PHM Editorial Board.
